# Access to mechanical thrombectomy and ischemic stroke mortality in Japan: a spatial ecological study

**DOI:** 10.3389/fneur.2023.1209446

**Published:** 2023-09-05

**Authors:** Kazuki Ohashi, Toshiya Osanai, Kensuke Fujiwara, Takumi Tanikawa, Yuji Tani, Soichiro Takamiya, Hirotaka Sato, Yasuhiro Morii, Katsuhiko Ogasawara

**Affiliations:** ^1^Faculty of Health Sciences, Hokkaido University, Sapporo, Japan; ^2^Department of Neurosurgery, Faculty of Medicine and Graduate School of Medicine, Hokkaido University, Sapporo, Japan; ^3^Graduate School of Commerce, Otaru University of Commerce, Otaru, Japan; ^4^Faculty of Health Sciences, Hokkaido University of Science, Sapporo, Japan; ^5^Department of Medical Informatics and Hospital Management, Asahikawa Medical University, Asahikawa, Japan; ^6^Department of Neurosurgery, Otaru General Hospital, Otaru, Japan; ^7^Department of Neurosurgery, Asahikawa Medical University, Asahikawa, Japan; ^8^Center for Outcomes Research and Economic Evaluation for Health, National Institute of Public Health, Wako, Japan

**Keywords:** mechanical thrombectomy, 2-step floating catchment area method, conditional autoregressive model, spatial accessibility, ischemic stroke

## Abstract

**Background:**

Advances in stroke treatment have greatly improved outcomes; however, disparities in access to treatment might increase. Achieving equitable access to stroke treatment is a health policy challenge, as rapid treatment is essential for positive outcomes. This ecological cross-sectional study aimed to determine the relationship between the disparities in spatial accessibility to mechanical thrombectomy (SAMT) and stroke mortality rates in Japan, hypothesizing that disparities in SAMT may increase the differences in stroke mortality between regions.

**Methods:**

We used the average number of ischemic stroke (IS) deaths between 2020 and 2021 as the response variable; and SAMT, medical resources, and socioeconomic characteristics of each municipality as explanatory variables. A conditional autoregressive model was used to examine the association between the risk of stroke mortality and SAMT. The standardized mortality ratio (SMR) was mapped to understand the nationwide disparities in stroke mortality risk.

**Results:**

The median number of IS deaths was 17.5 persons per year in the municipalities (2020 to 2021). The study also found that municipalities with low SAMT were located in the northern part of Japan. The non-spatial regression model results indicated that poor accessibility, a small proportion of bachelor’s degrees or higher, and a high proportion of workers in secondary industries were related to high IS mortality. Three models were evaluated using spatial analysis; Model 1 with accessibility indicators alone, Model 2 with medical resources added to Model 1, and Model 3 with socioeconomic characteristics added to Model 2. In Models 1 and 2, the population-weighted spatial accessibility index (PWSAI) showed a significant negative relationship with stroke mortality. However, this was not evident in Model 3. Mapping using Model 3 showed that the high-risk areas were predominantly located in northern Japan, excluding Hokkaido.

**Conclusion:**

Access to mechanical thrombectomy was estimated, and regional differences were observed. The relationship between accessibility and IS mortality is unknown; however, regardless of accessibility, municipalities with a high proportion of workers in secondary industries and a small proportion with bachelor’s degrees or above are at risk of death from stroke.

## 1. Introduction

Stroke is the second leading cause of death, with approximately 6.55 million deaths worldwide in 2019 (1). More than 100,000 people die annually from a stroke in Japan, and ischemic stroke (IS) accounts for 60% (2). Advances in the treatment of stroke, including intravenous thrombolysis (IVT) and mechanical thrombectomy (MT), greatly improved stroke outcomes (3–5). Meanwhile, the advancement and diffusion of treatment depend on the expertise of physicians and hospitals, which may increase disparities in access to treatment (6, 7). Achieving equitable access is a health policy challenge because stroke outcomes depend on access to the right and rapid treatment. In Europe, disparities in access to IVT and MT between countries have emerged, and targets have been declared to be reached by 2030 for the implementation rate of each treatment to reduce these disparities (8, 9). In the United States, primary and comprehensive stroke centers established in the early 2000s have improved the quality and accessibility of stroke care and overall stroke outcomes (10, 11). Following the leadership of the U.S., the Japan Stroke Society aims to improve the quality of stroke care and achieve equitable access by certifying primary stroke centers (PSCs) (12). However, Maeda et al. revealed geographical disparities in the MT receiving rates among prefectures (6). These disparities may cause regional disparities in stroke mortality; there is no evidence of a relationship between the disparities in spatial accessibility to MT (SAMT) and stroke mortality. In countries with large differences in population density (e.g., the U.S., Canada, and Australia), there is much evidence of disparities in spatial access to acute stroke treatment (7, 13, 14). On the other hand, there is little evidence from smaller countries such as Japan. Moreover, In Japan, there is a mix of municipalities with large differences in population density, making it difficult to clearly divide all municipalities into urban and rural areas. In other words, municipalities defined as rural in terms of population density may also be adjacent to urban areas. Estimating accessibility in this study using geographic information systems overcomes this challenge.

Typical indicators of accessibility and availability of emergency care and healthcare include the number of physicians and hospitals per population, distance to the nearest facility, and the gravity model (15, 16). Among these, the two-step floating catchment area (2SFCA) method proposed by Luo and Wang is commonly used because it can capture more realities by including the demand, supply, and distance/time factors (17). Amiri et al. used the 2SFCA to quantify access to primary care physicians in Washington State and demonstrated that areas with poor access to primary care physicians had higher all-cause, cancer, and heart disease mortality rates (18). In contrast, Bauer et al. revealed that travel time to the nearest hospital was a more suitable indicator of accessibility to acute stroke treatment than the 2SFCA method (19). Thus, this study employed two indicators to quantify the SAMT: the spatial accessibility index (SAI) using the 2SFCA method and travel time to the nearest hospital. The 2SFCA method considered the demand for MT, supply, and travel time to facilities. A distance decay function was applied to conceptualize moving to an MT-feasible facility with a shorter travel time when multiple facilities could be selected (20, 21). To the best of our knowledge, the relationship between SAMT and IS mortality remains poorly understood. This study hypothesized that disparities in SAMT increase the differences in stroke mortality between regions and aimed to determine the relationship between SAMT and stroke mortality at the municipal level, the smallest administrative unit in Japan. This study provides a rationale for healthcare policies that increase SAMT and specific areas where intervention is needed, as well as to support decision-making by healthcare policymakers.

## 2. Materials and Methods

### 2.1. Study target

The object of this research was municipalities, and the 23 wards of Tokyo and ordinance-designated cities were dealt with by the ward units that constituted them. This was the minimum unit for official statistics regarding the number of deaths due to IS. First, remote islands (63 municipalities) with no adjacent municipalities were excluded because the conditional regression model adopted in this study assumes that all samples have at least one adjacency relationship. Furthermore, six municipalities in Fukushima Prefecture were designated as evacuation zones in 2015 because of the impact of the Great East Japan Earthquake. They had zero or very few populations removed. Finally, 1827 municipalities were analyzed. The number of IS deaths in these municipalities in 2021 was 99.3% of total deaths in Japan (2).

### 2.2. Study design

This was an ecological cross-sectional study using the number of IS deaths (ICD-10: I63 and I69.3) in municipalities, the smallest administrative units in Japan. We adopted the response variable, the average number of stroke deaths per municipality (2020 and 2021), and the explanatory variables, the population-weighted SAI (PWSAI), the population-weighted travel time (PWTT) to nearest MT capable hospital, medical resources [number of physicians (2018), general hospitals (2019), clinics (2019), and emergency hospitals (2019); all per 10,000 population], socioeconomic characteristics [proportion of workers in primary (e.g., agriculture and fishery), secondary (e.g., blue-collar workers), and tertiary (e.g., white-collar workers) industries] (22, 23), and proportion of bachelor’s degree or above. Individual-level socioeconomic status and level of community deprivation have been found to be associated with stroke incidence and death from stroke (24–26). Therefore, these explanatory variables of municipalities may explain the disparities in the risk of stroke mortality. There were no data on other socioeconomic characteristics such as mean individual income or official poverty rate for each municipality. Moreover, the unemployment rate was excluded in this study because Japan’s unemployment rate has remained low at 2.0–5.4% from the 1980s to 2020s (27), mean unemployment rate among this study area was 3.7% (±1.1) in 2020 (28), so we judged that the impact could be ignored. To adjust for the population size, the expected number of stroke deaths in each municipality was used as an offset variable, referring to the number of IS deaths nationwide. The expected number of IS stroke deaths was calculated and summed by sex and age group in 5-year increments. Using these variables, we constructed a model to explain the number of stroke deaths and examined the association between the risk of stroke death and the SAMT. We also calculated the standardized mortality ratio (SMR) using the ratio of the number of deaths estimated by the model to the expected number of deaths and estimated the risk of stroke mortality at the municipal level.

### 2.3. Data source

The number of IS deaths was obtained from Vital Statistics by municipality, sex, and age (from 0 to 79 years old in 5-year increments, and 80 years old and over) (2), corresponding to the selected cause of death classification Se-24. This corresponds to ICD-10 codes I63 and I69.3. The national population data for calculating each municipality’s expected deaths and socioeconomic characteristics were obtained from the 2020 census (28). All medical resource data were obtained from the e-Stat (29). Geographic information (population, medical facilities, 500 m*500 m mesh) stored in a shapefile was obtained from the National Land Numerical Information Download Service (30), and road data were obtained from the ArcGIS Geo Suite Road Network 2021 (Esri Japan, Sumitomo Electric, Tokyo, Japan). PSCs in 2021 and neurointerventionalists in 2021 were obtained from the websites of the Japanese Stroke Association and the Japanese Society for Endovascular Therapy, respectively, as sources of stroke treatment (31, 32). Among PSCs, hospitals with neurointerventionalists were defined as MT-capable hospitals. However, there was no data on the number of actual MT implementations. The distance between each mesh and PSC was calculated using ArcGIS Pro 3.0 (ESRI, Redlands, CA, USA, https://www.esri.com/en-us/home). Other analyses and drawings were performed in R4.2.0 (33) and R studio (34) and the R package “CARbayes” (35).

### 2.4. Access to mechanical thrombectomy at municipality

**Figure 1:**
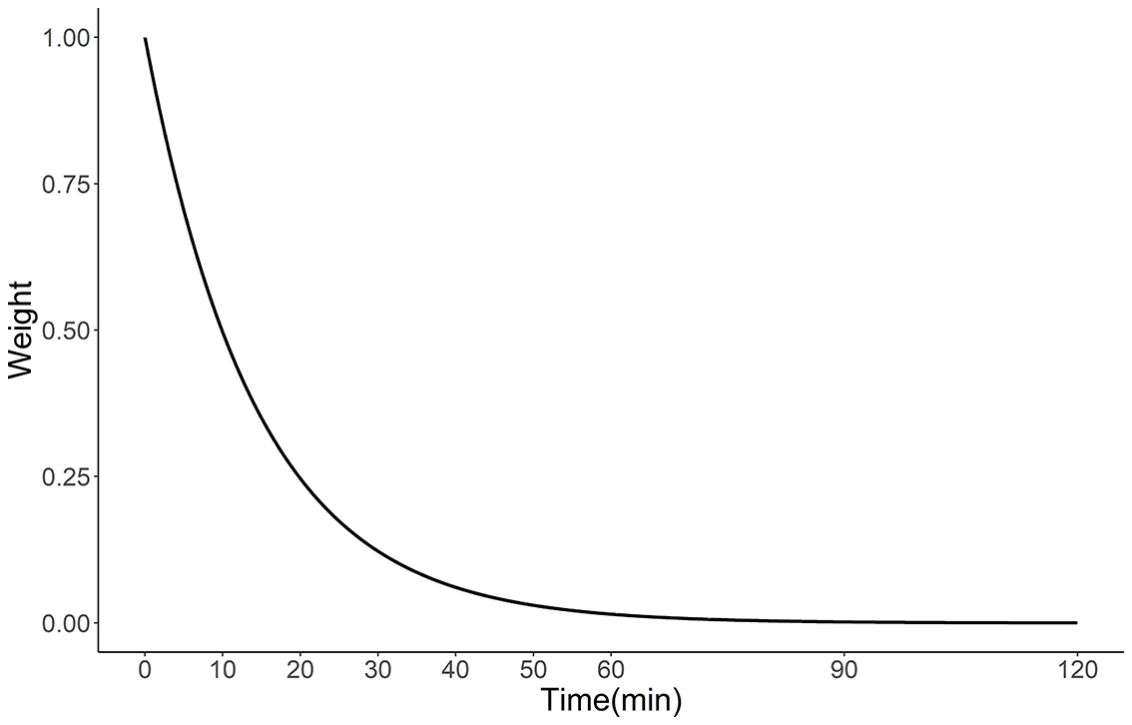
Distance decay function obtained using Eq. 3 (β = 0.07).

To calculate the SAI, the estimated population aged 65 and over in 2020 was created based on the 2015 census included in the 500-meter mesh data (demand), and 661 MT-capable facilities (Supplementary 1) with 1,604 neurointerventionalists in the facilities (supply) were incorporated into the 2SFCA method. One hospital was excluded because the hospital was located on an island without an adjacent municipality. The 500-meter mesh data, the smallest grid data for the estimated population, was opened by the Ministry of Land, Infrastructure (30). We, therefore, concluded that using this mesh data would provide an accurate estimate of accessibility. We created a travel time matrix based on travel time by car between each mesh and facility. The 2SFCA method consists of a two-step process. In the 1st step, the population aged 65 and over in the mesh within a 120-min range from each hospital was totaled and divided by the number of neurointerventionalists enrolled, and the demand-to-supply ratio was calculated for each hospital (Eq. 1). Second, the hospital’s demand-to-supply ratio within 120 min starting from each mesh was totaled to obtain the SAI for each mesh (Eq. 2). A 120-min range, distance decay function (Figure 1), and friction coefficient (Eq. 3) were assumed based on previous studies; Japan’s emergency medical system transported about 99% of the patients to the hospital within 2 h (36), the median transport time for patients undergoing mechanical thrombectomy was 10 min (37). The SAI tied to each mesh was converted into the PWSAI (Eq. 4), an accessibility variable per municipality (38). This corresponds to the number of neurointerventionalists *per capita*, considering the mobility across administrative boundaries.


(1) $$r_{j}=\frac{S_{j}}{\sum_{d_{ij}\in d_{0}}D_{i}*f\left(d_{ij}\right)}$$

(2) $$A_{i}=\sum_{d_{ik}\in d_{0}}r_{k}*f\left(d_{ik}\right)$$

(3) $$f\left(d_{ij}\right)=\left\{\begin{array}{c} e^{-\beta *d_{ij}},\;d_{ij}<d_{0}\;\\ \;\\ 0,\;d_{ij}\geq d_{0} \end{array}\right.$$

(4) $$\mathrm{PWSAI}=\frac{\sum_{i\in I}D_{i}*A_{i}}{\sum_{i\in I}D_{i}}$$

where i is mesh, k, j is hospital, r is demand/supply ratio at facility, S is the number of neurointerventionalists in each facility, β is friction coefficient (=0.07 in this study), dij, dik is the travel time between mesh i and hospital j or k, d0 is reachable area (within 120 min), A is the spatial accessibility index, D is the population over 65 years of age, and I is municipality.

PWTT is a simpler indicator. The total travel time from each location to the nearest facility, multiplied by the estimated population of each location, was divided by the population of the entire municipality (Eq. 5).


(5) $$\mathrm{PWTT}=\frac{\sum_{i\in I}D_{i}*T_{ik}}{\sum_{i\in I}D_{i}}$$

where i is the mesh, k is the hospital, D is the population over 65 years of age, T is the travel time from mesh i to hospital k, and I is the municipality.

### 2.5. Statistical analysis

First, a non-spatial generalized linear model based on a negative binomial distribution (Eq. 6) was run to check for multicollinearity among the explanatory variables and to exclude variables with a variance inflation factor (VIF) > 5 (39). The residuals of the non-spatial generalized linear model were then tested using Moran’s I statistic (40) to confirm their spatial autocorrelation. The Bayesian Conditional Autoregressive model (Eq. 7), a generalized linear mixed model capable of dealing with spatial autocorrelation, was then applied and analyzed as a Poisson distribution. The Leroux model was employed to derive spatial autocorrelation (41, 42). This Bayesian model can account for the uncertainty in smaller municipalities (42). Similarly, Moran’s I test was applied to the residuals of the spatial regression models to confirm that spatial autocorrelation has been removed. Model 1 was an accessibility model including the PWSAI and PWTT, Model 2 was a medical resource model with the addition of variables related to medical resources to Model 1, and Model 3 was a full model with all variables adjusted. The results for each model show the posterior median relative risk per standard deviation and 95% credible interval (Cr). The goodness of fit of the models was evaluated using the Watanabe–Akaike information criterion (WAIC) (43), and the estimated SMR was calculated using the best-fitting model and mapped at the municipal level as the risk of IS death. The model parameters were estimated via Bayesian estimation using Markov-chain Monte Carlo (MCMC) simulations. MCMC was performed by creating three independent chains, each of which was sampled 52,0000 times, including 20,000 burn-in periods, and thinned every 100 times to create 15,000 samples. The convergence of MCMC stationarity was considered to have been achieved if the Gelman-Rubin potential scale reduction factor was less than 1.1 (44).


$$O_{i}\sim NB\left(r,E_{i}\theta_{i}\right)$$

(6) $$\log\left(\theta_{i}\right)=\mathrm{offset}\left(\log E_{i}\right)+\alpha +\beta X_{i}$$

$$O_{i}\sim \mathrm{Poisson}\left(E_{i}\theta_{i}\right)$$

$$\log\left(\theta_{i}\right)=\mathrm{offset}\left(\log E_{i}\right)+\alpha +\beta X_{i}+\psi_{i}$$

$$\psi_{i}\sim N\left(\frac{\rho \sum_{l=1}^{n}w_{il}\psi_{l}}{\rho \sum_{l=1}^{n}w_{il}+1-\rho },\frac{\tau^{2}}{\rho \sum_{l=1}^{n}w_{il}+1-\rho }\right)$$

$$\tau^{2}\sim \text{Inverse}-\text{Gamma}\;\left(1,0.01\right)$$

(7) $$\rho \sim \text{Uniform}\;\left(0,1\right)$$

where, O, and E are the number of IS deaths and expected deaths in each municipality, θ is the relative risk of IS, α is intercept, and β is the coefficient of variable X. ψ is the spatial structure random effect via Leroux model (41, 42). W_il_ is a binary adjacency matrix of the queen type for the adjacencies of municipalities i and l. Additionally, the definition of adjacency was used for traffic on bridges accessible to automobiles. The prior distribution for $\tau^{2}$ was given as inverse-gamma (1, 0.01); the prior distribution for ρ was given as Uniform (0,1). This process was performed in the main analysis for total stroke deaths and by gender as a sub-analysis same explanatory variables were used as in the main analysis. To check the effect of the prior distribution of $\tau^{2}$, a sensitivity analysis was performed by changing the prior distribution to inverse gamma (1, 0.01) and (0.5, 0.005).

### 2.6. Ethical statement

This ecological study used public rather than individual data. Therefore, ethics committee approval was not required for this study.

## 3. Results

### 3.1. Descriptive statistics of the variables and exploratory analyses

**Figure 2:**
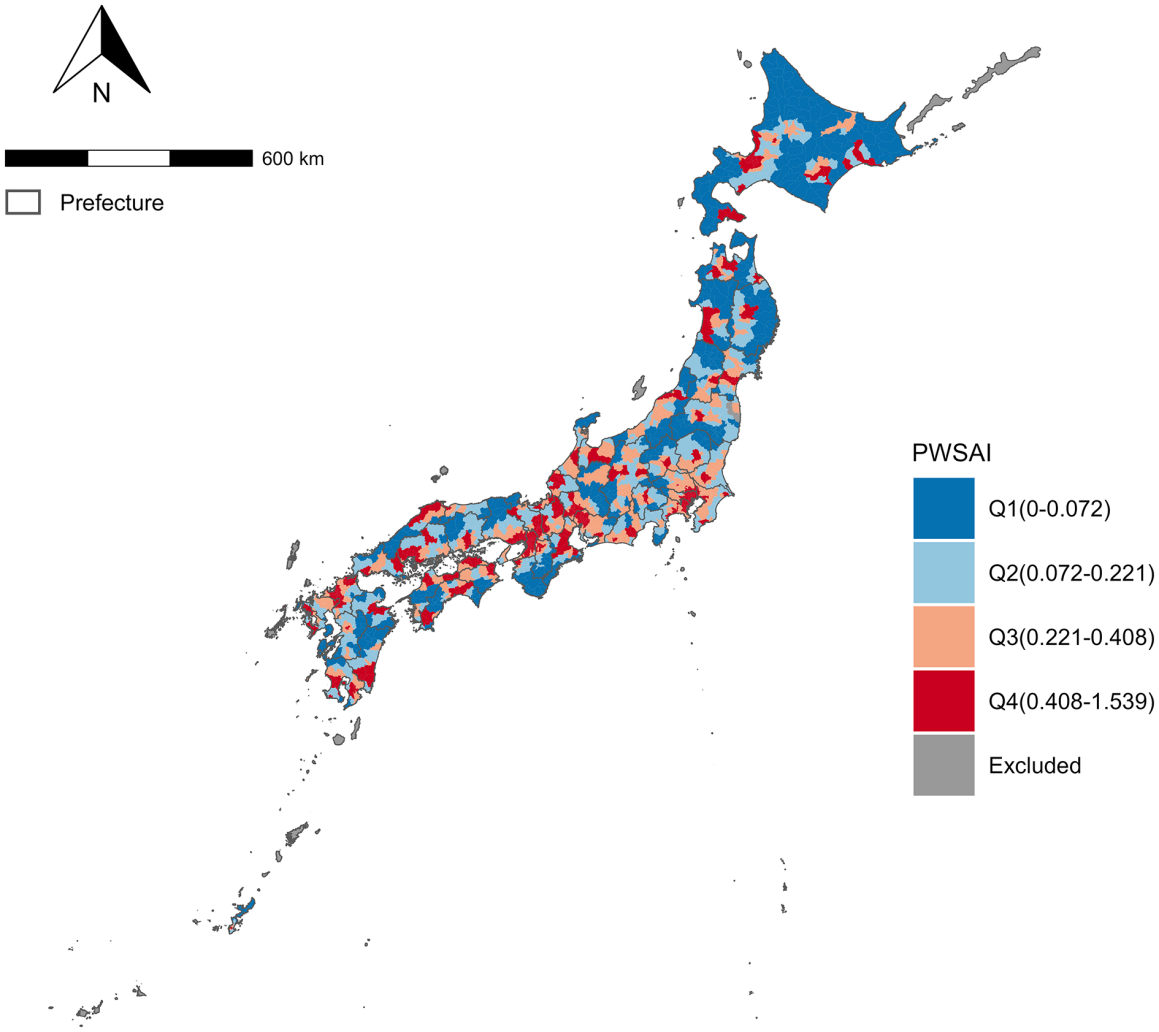
Population-weighted spatial accessibility index of Mechanical thrombectomy at Municipality in Japan. PWSAI, Population-weighted spatial accessibility. A choropleth map of the PWSAI divided into quadrant ranges. Grayed areas were excluded from the analysis.

All variables in this study are described in Table 1. The median number of IS deaths was 17.5 in these municipalities. PWSAI was median 0.22 [IQR: 0.06–0.42], median PWTT was 0.38 [IQR: 0.22–0.71]. Municipalities with a low SAMT emerged in northern Japan (Figure 2).

**Table 1:** Overview of each parameter at municipalities.

	Median [IQR]	Mean [SD]	Range
Number of ischemic stroke deaths (2020 to 2021)	17.5 [6.8–41.3]	31.3 [39.2]	0–309
Men	8.0 [3.0–19.0]	15.1 [19.4]	0–153.5
Women	9.0 [3.5–21.0]	16.3 [20.2]	0–155.5
Population (2020)	29,320 [9180–82,454]	66,235 [94932]	351–916,972
Men	14,177 [4434–40,124]	32,191 [46234]	171–432,101
Women	15,108 [4745–41,971]	34,044 [48753]	177–484,871
Inhabitable area (km^2^)	41.6 [20.37–84.97]	65.8 [69.8]	1.5–805.24
Population density (/km^2^)	552.5 [249.2–1710.3]	1882.6 [3213.1]	9.6–20,911
PWSAI	0.22 [0.06–0.42]	0.28 [0.27]	0.0–1.9
PWTT (hour)	0.38 [0.22–0.71]	0.55 [0.49]	0.1–4.2
Number of physicians^*^	14.8 [8.7–22.4]	19.3 [21.0]	0.0–275.7
Number of hospitals^*^	0.5 [0.2–0.9]	0.7 [0.7]	0.0–7.7
Number of clinics^*^	7.5 [6.0–9.3]	8.2 [4.6]	0.0–78.4
Number of emergency hospitals^*^	0.3 [0.0–0.5]	0.4 [0.6]	0.0–6.1
Workers in primary industry (%)	6.1 [1.9–14.1]	9.4 [9.8]	0.0–75.9
Workers in secondary industry (%)	24.8 [19.0–30.6]	24.8 [8.1]	2.3–53.5
Workers in tertiary industry (%)	64.6 [57.8–73.3]	65.4 [10.6]	20.7–93.5
Bachelor’s degree or above (%)	14.2 [10.4–19.4]	15.8 [7.4]	3.5–47.8

PWSAI, population-weighted spatial accessibility index; PWTT, population-weighted travel time to the nearest MT capable hospital; IQR, interquartile range; SD, standard deviation; Range; Min – Max.

* Per 10,000 population.

Workers in the tertiary industry were excluded because the VIF was >5 after an explanatory analysis of all variables. In the non-spatial regression model, the coefficients for the PWSAI and bachelor’s degrees or above were negative. The coefficients of the number of physicians (per 10,000) and workers in the secondary industry (%) were positive. Thus, poor accessibility, a small proportion of bachelor’s degrees or above, and a high proportion of workers in secondary industries were related to high IS mortality (Table 2). However, Moran’s I statistics indicated a significant spatial autocorrelation of the residuals (I = 0.216, *p* < 0.001), highlighting the need for spatial analysis.

**Table 2:** Result of non-spatial regression model.

	Coefficient	95%CI	*p* value	VIF
Intercept	0.174	0.076, 0.272	<0.001	
PWSAI	−0.077	−0.143, −0.012	0.021	2.592
PWTT (hour)	−0.006	−0.051, 0.039	0.796	2.079
Number of physicians^*^	0.001	0.000, 0.001	0.079	1.652
Number of hospitals^*^	−0.025	−0.058, 0.007	0.130	2.254
Number of clinics^*^	0.000	−0.004, 0.004	0.871	1.455
Number of emergency hospitals^*^	0.018	−0.028, 0.064	0.435	2.102
Workers in primary industry (%)	0.001	−0.001, 0.004	0.232	2.248
Workers in secondary industry (%)	0.004	0.002, 0.006	<0.001	1.602
Bachelor’s degree or above (%)	−0.013	−0.015, −0.010	<0.001	2.558
Moran’s I statistics	0.219		<0.001	

PWSAI, population-weighted spatial accessibility index; PWTT, population-weighted travel time to the nearest MT capable hospital; CI, confidence interval; VIF, variance inflation factor.

* Per 10,000 population.

### 3.2. Results of spatial analyses

**Figure 3:**
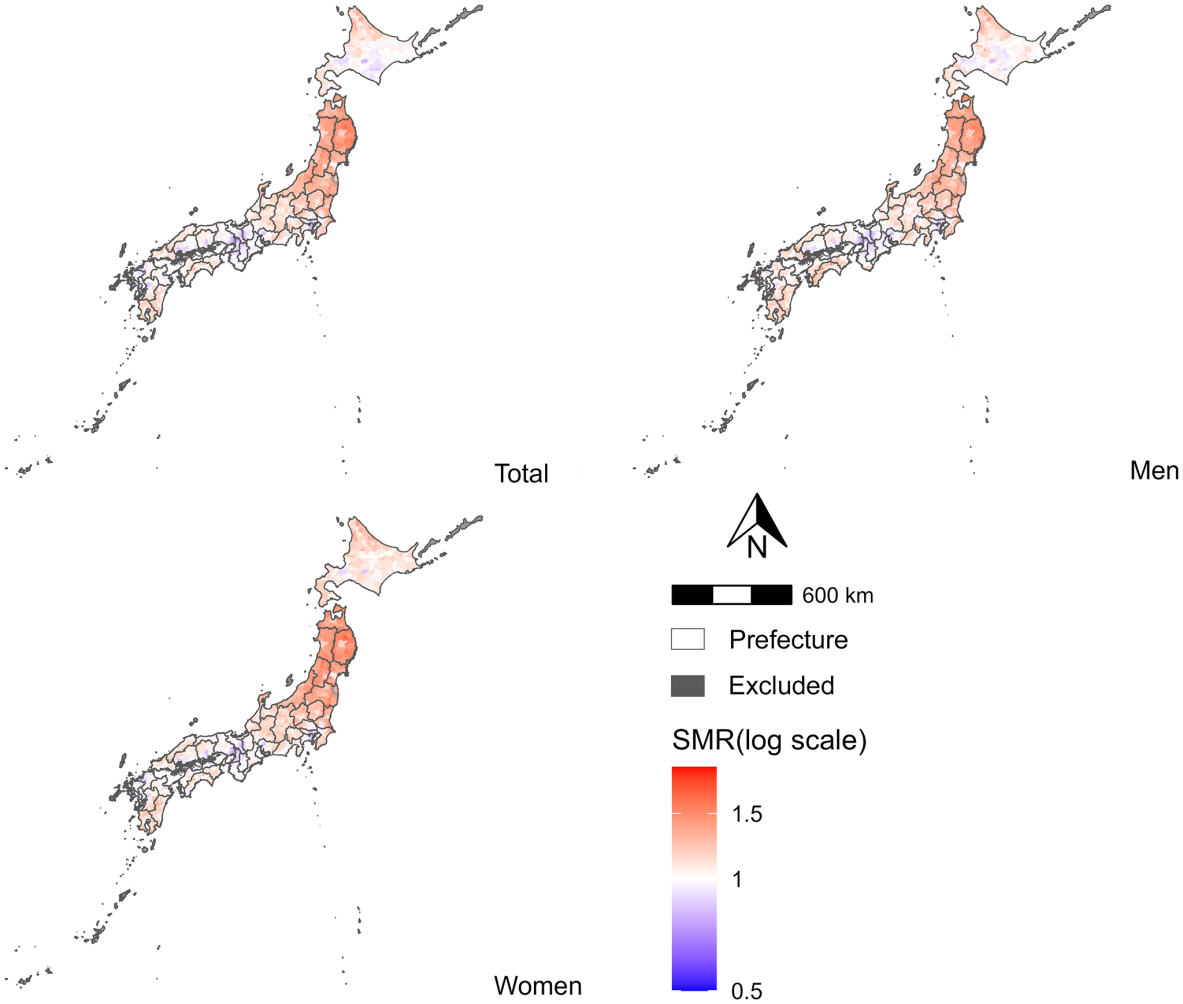
Distribution of the estimated mortality risk of ischemic stroke in Japan. IS standard mortality ratio in each municipality. Median estimated SMR (95% Cr) was 1.04 (0.91–1.20) in total, 1.06 (0.95–1.21) in men, and 1.07 (0.96–1.22) in women.

Table 3 summarizes the results of the three models. All coefficients were transformed into the relative risk (RR) per standard deviation. Models 1 and 2 showed that high PWSAI (Model 1: RR = 0.971, 95% Cr = 0.954–0.988; Model 2: RR = 0.978, 95% Cr = 0.960–0.997) and low PWTT (Model 1: RR = 1.027, 95%Cr = 1.002–1.053; Model 2: not significant) were associated with low IS mortality. Municipalities with a PWSAI of 1SD higher had a 2.2–2.9% lower risk of IS mortality, and those with a PWTT of 1SD had a 2.7% higher risk of IS mortality. In contrast, these variables were not significantly associated with mortality after adjusting for socioeconomic characteristics in the full model. This model showed that the proportion of workers in secondary industries and the proportion with a bachelor’s degree or above were significant variables for IS mortality. None of the models had a significant Moran’s I. Model 3 was the best-fitting model, based on the lowest WAIC. In the sub-analysis, the proportion of workers in secondary industries and the proportion of those with a bachelor’s degree or above were also significant variables for the risk of stroke mortality in both men and women. On the other hand, the risk of stroke mortality was higher for men with a higher PWSAI and several hospitals and clinics (Supplementary 1). Median estimated SMR (95%Cr) was 1.04 (0.91–1.20) in total, 1.06 (0.95–1.21) in men, and 1.07 (0.96–1.22) in women. SMR at each municipality as IS mortality was mapped using Model 3, and high mortality was observed in northern Japan, excluding Hokkaido (Figure 3).

**Table 3:** Comparison of three spatial models in total population.

	Model 1		Model 2		Model 3	
	RR	95%Cr	RR	95%Cr	RR	95%Cr
PWSAI	0.971	0.954–0.988	0.978	0.960–0.997	1.012	0.994–1.031
PWTT (hour)	1.027	1.002–1.053	1.024	0.999–1.050	1.013	0.990–1.036
Number of physicians^*^			0.987	0.974–1.000	1.001	0.988–1.013
Number of hospitals^*^			1.028	1.003–1.054	1.022	0.998–1.047
Number of clinics^*^			0.997	0.980–1.015	1.015	0.998–1.032
Number of emergency hospitals^*^			1.007	0.981–1.033	1.003	0.978–1.028
Workers in primary industry (%), 2020					1.010	0.986–1.034
Workers in secondary industry (%), 2020					1.028	1.010–1.046
Bachelor’s degree or above (%), 2020					0.916	0.898–0.934
WAIC (chain 1)	10,427		10,423		10,382	
Moran’s I (chain 1)	−0.028		−0.028		−0.018	

PWSAI, population-weighted spatial accessibility index; PWTT, population-weighted travel time to the nearest MT capable hospital; WAIC, Watanabe–Akaike information criterion; RR, relative risk; Cr, credible interval. Model 1; included spatial accessibility factors, Model 2; Model 1 + medical resource factors, Model 3; Model 2 + socioeconomic factors.

* Per 10,000 population.

### 3.3. Evaluation of MCMC convergence for models 1–3 and sensitivity analysis

All models in the main analysis obtained a Gelman–Rubin potential scale reduction factor of <1.1, and the MCMC converged. Sensitivity analysis for Model 3 was performed using two different prior distributions, and both yielded the same results as those obtained in the first analysis (Supplementary 1).

## 4. Discussion

Our findings suggest that access to MT, one of the main treatment modalities for acute stroke, may be associated with regional disparities in the stroke mortality risk at the first time. At the municipal level, the smallest administrative unit in Japan, our results imply that areas with better access to mechanical thromboprophylaxis may have a lower risk of stroke (Models 1 and 2). However, the scope of these findings is limited. Contrary to our hypothesis, Model 3 showed that the PWSAI and PWTT were not significantly related to IS mortality after adjusting for socioeconomic characteristics. Furthermore, for men, a significant positive relationship existed between better PWSAI and higher mortality. In all analyses, the proportion of workers in the secondary industry and those with a bachelor’s degree or higher were significantly related to IS mortality. The low socioeconomic status of individuals (e.g., education, income, and occupation) was strongly related to high mortality or a high incidence of stroke (45–47), which is consistent with previous results. In Japan, smoking habits decrease in individuals with a higher educational background (48, 49), and it can be inferred that the IS incidence rate decreases in municipalities with a higher proportion of individuals with bachelor’s degrees or above. To summarize, access to acute stroke care is important, and its extent varies by region. In regions with low accessibility, achieving equitable access is a future challenge. For example, access can be enhanced by promoting the tele-stroke system as a healthcare policy (50, 51). However, our findings suggest that increasing accessibility alone may not be sufficient to reduce the risk of stroke mortality. Considering the socioeconomic characteristics of municipalities, certain actions, such as reducing hypertension and smoking, can greatly contribute towards stroke prevention and improving stroke outcomes.

We analyzed access to MT. First, PWSAI and PWTT were negatively and positively correlated with IS mortality, respectively (model 1 and model 2), consistent with the cohort study that showed that stroke mortality increased with longer transport time to the emergency hospital (52). This is intuitive and reasonable. Short supply due to travel time and increased travel time to specialty facilities were associated with worse mortality. This study measured a more realistic SAI incorporating demand, supply, and travel time. Although there are non-spatial accessibility barriers to medical care, such as insurance and income (53), Japan has a universal health insurance system. Disparities in access to emergency care due to differences in insurance and income are unlikely to occur, and standard emergency care is available for small copayments (54). In other words, the main barriers to accessing emergency care were spatial factors. One spatial factor in emergency care access is the performance of emergency transport systems. With an average time of hospital arrival of approximately 40 min (36), these systems contribute to the time window of acute IS (4.5 or 8–24 h) (55). Moreover, PSCs capable of performing intravenous thrombolysis, another main treatment for acute IS, have been established nationwide, and 98.9% of the population can access intravenous thrombolysis within 1 h (56). This fact suggests that a robust ambulance transport system and widespread adoption of intravenous thrombolysis may suppress death from IS regardless of access to MT. In the future, studies on other outcomes, such as sequelae and severity, are needed to understand the influence of access to stroke treatment.

More advanced treatments for acute ischemic stroke have been performed in prefectures with a high population density in Japan (6), similar to that observed in the United States. Timely access to appropriate stroke care is more common in urban areas than rural areas (57). In urban areas of Japan, the population is highly educated, and its industrial structure has shifted from secondary to tertiary industries (28). Therefore, it can be assumed that many municipalities with higher socioeconomic characteristics and lower IS mortality rates have good access to healthcare, which may be part of the mechanism underlying the association between an individual’s socioeconomic status and mortality risk. However, the present study did not provide sufficient evidence to support this finding. In the future, a multilevel analysis combining personal data and geographic information will be required to clarify the relationship between SAMT and stroke mortality.

The 2SFCA method, which applies an exponential distance decay function, was used to evaluate the SAMT. The distance decay function and friction coefficient were extrapolated based on previous studies. To capture more realistic accessibility using this method, detailed data on the transportation of stroke patients are required. Furthermore, there was insufficient agreement between the threshold values obtained using this method. Ensuring the validity and reliability of the accessibility index will be a challenge in the future.

### 4.1. Limitations

Our study had several limitations. First, this ecological study focused on municipalities and limited access to several socioeconomic factors were available. Therefore, the possibility of an ecological fallacy is unavoidable, and uncontrolled socioeconomic factors may influence the results. Second, this study did not consider the impact of COVID-19. COVID-19 caused the number of hospitalizations for stroke to decrease during its spread (58), and there is a likelihood of a disparity in the spread effect on our results. The incidence of acute IS by region was also unknown. Therefore, we assumed that the number of stroke cases was defined by population size. However, this assumption may have caused the difference in results between men and women, which is a topic for the future work. Third, the selection of variables required to estimate accessibility via the 2SFCA depended on the researcher. The demand, supply, and distance decay functions used in the estimation were based on previous studies; however, their validity could not be confirmed. Using real-world data to demonstrate the validity of SAI will advance future research. Fourth, the lifestyle factors data related to stroke incidence (e.g., smoking, alcohol, diet, exercise) were not used in this study because there were no data on the municipality level. These data may contribute to clarify the difference of result between men and women in sub analysis. Finally, deaths in this study included both acute and chronic ischemic strokes. Therefore, it may be possible to prove our hypothesis using only acute ischemic stroke, which is more strongly affected by MT. However, a special application of such data is required.

## 5. Conclusion

Large municipalities in northern Japan had low SAMT, and IS mortality was high, with the exception of Hokkaido. However, there was no significant association between the SAMT and IS mortality after adjusting for the socioeconomic characteristics of the municipality. Areas with a high proportion of secondary industry workers and a low proportion of the population with a bachelor’s degree or higher are likely to have higher IS mortality, regardless of SAMT.

## Data availability statement

The raw data supporting the conclusions of this article will be made available by the authors, without undue reservation.

## Author contributions

KazO, TO, and KatO concepted the study design. KazO analyzed the results and wrote the manuscript. KF, TT, YT, ST, HS, and YM revised the article critically and interpreted the data. All authors contributed to the article and approved the submitted version.

## Conflict of interest

The authors declare that the research was conducted without any commercial or financial relationships that could be construed as a potential conflict of interest.

## Publisher’s note

All claims expressed in this article are solely those of the authors and do not necessarily represent those of their affiliated organizations, or those of the publisher, the editors and the reviewers. Any product that may be evaluated in this article, or claim that may be made by its manufacturer, is not guaranteed or endorsed by the publisher.

## Abbreviations

2SFCA: The two-step floating catchment area
Cr: Credible interval
IS: Ischemic stroke
IVT: Intravenous thrombolysis
MCMC: Markov-chain Monte Carlo
MT: Mechanical thrombectomy
PSCs: Primary stroke centers
PWSAI: Population-weighted spatial accessibility index
PWTT: Population-weighted travel time
RR: Relative risk
SAI: Spatial accessibility index
SAMT: Spatial accessibility to mechanical thrombectomy
SMR: Standardized mortality ratio
WAIC: Watanabe–Akaike information criterion
