# Supplementary material for: Access to mechanical thrombectomy and ischemic stroke mortality in Japan: a spatial ecological study
Source: Front Neurol. 2023 Sep 5;14:1209446. doi: 10.3389/fneur.2023.1209446 (PMC10507726; doi:10.3389/fneur.2023.1209446)
Supplement: Supplementary file 1 [file Data_Sheet_1.docx]

Figure S1 Distribution of MT-capable hospital in this study


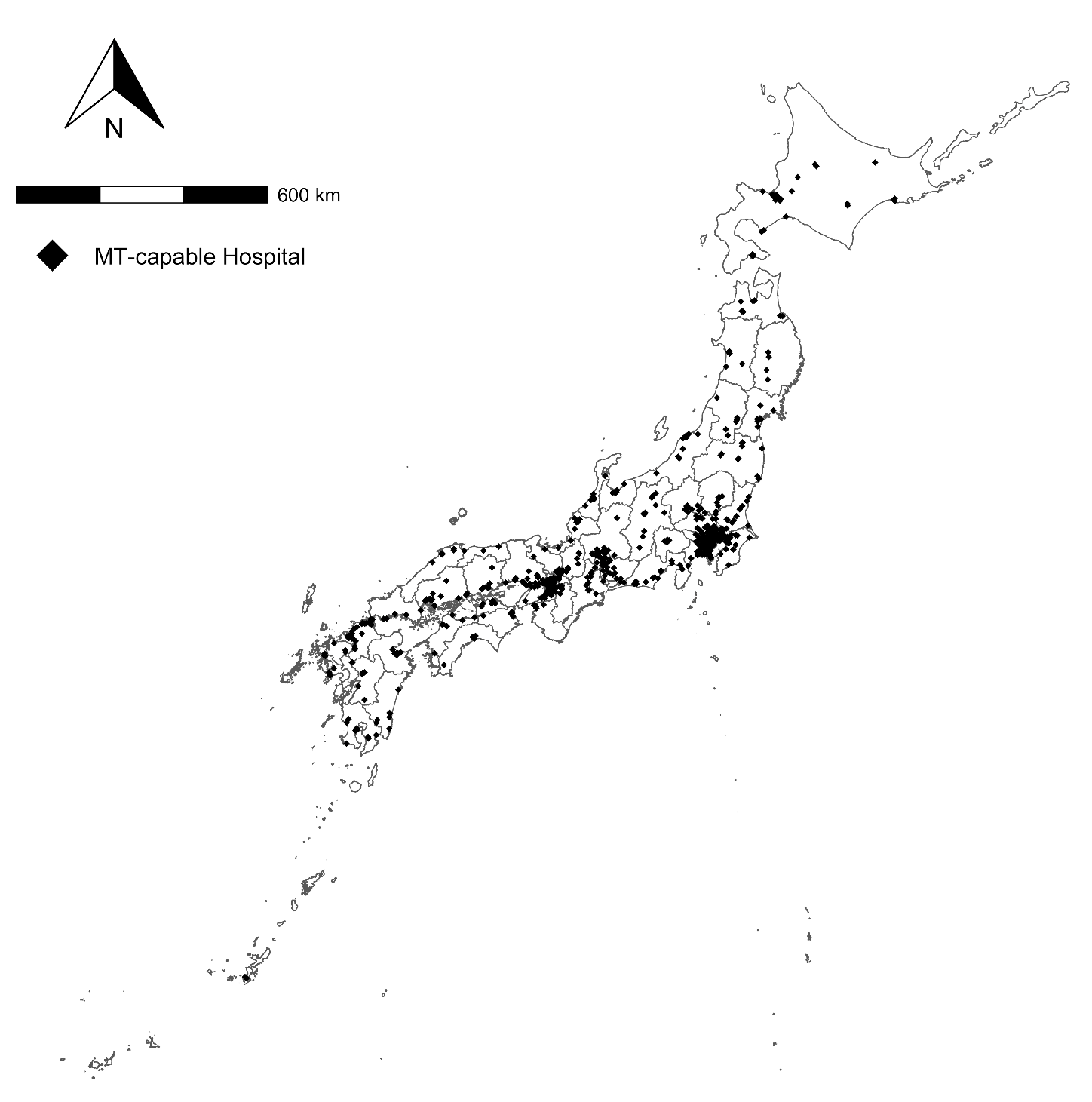


This study enrolled 661 hospitals as MT-capable hospitals. One hospital was excluded because of the hospital locate in island without adjacent municipality. MT; Mechanical thrombectomy.

Table S1 Result of three spatial models in men.

|  | Model 1 | | Model 2 | | Model 3 | |
| --- | --- | --- | --- | --- | --- | --- |
|  | RR | 95%Cr | RR | 95%Cr | RR | 95%Cr |
| PWSAI | 0.981 | 0.961-1.002 | 0.988 | 0.966-1.011 | 1.027 | 1.004-1.050 |
| PWTT (hour) | 1.038 | 1.007-1.069 | 1.032 | 1.002-1.064 | 1.021 | 0.992-1.051 |
| Number of physicians* |  |  | 0.979 | 0.962-0.997 | 0.995 | 0.978-1.011 |
| Number of hospitals* |  |  | 1.064 | 1.028-1.100 | 1.050 | 1.015-1.085 |
| Number of clinics* |  |  | 1.007 | 0.984-1.031 | 1.029 | 1.006-1.052 |
| Number of emergency hospitals* |  |  | 0.985 | 0.951-1.020 | 0.981 | 0.947-1.015 |
| Workers in primary industry (%) |  |  |  |  | 0.995 | 0.963-1.027 |
| Workers in secondary industry (%) |  |  |  |  | 1.022 | 1.000-1.046 |
| Bachelor's degree or above (%) |  |  |  |  | 0.897 | 0.875-0.918 |
| WAIC（chain 1） | 8641.0 | | 8633.3 | | 8549.9 | |
| Moran’s I (chain 1) | 0.029** | | 0.029** | | 0.029** | |

PWSAI; population-weighted spatial accessibility index, PWTT, population-weighted travel time to the nearest MT capable hospital, WAIC; Watanabe–Akaike information criterion, Cr; credible interval, RR; relative risk, *per 10000 population, ** p<0.05

Model 1; included spatial accessibility factors, Model 2; Model 1 + medical resource factors, Model 3; Model 2 + socioeconomic factors.

Table S2 Result of three spatial models in women.

|  | Model 1 | | Model 2 | | Model 3 | |
| --- | --- | --- | --- | --- | --- | --- |
|  | RR | 95%Cr | RR | 95%Cr | RR | 95%Cr |
| PWSAI | 0.954 | 0.935-0.974 | 0.958 | 0.937-0.979 | 0.997 | 0.975-1.018 |
| PWTT (hour) | 1.025 | 0.996-1.055 | 1.020 | 0.992-1.051 | 1.005 | 0.978-1.033 |
| Number of physicians* |  |  | 0.994 | 0.977-1.011 | 1.009 | 0.993-1.025 |
| Number of hospitals* |  |  | 1.019 | 0.987-1.051 | 1.013 | 0.982-1.044 |
| Number of clinics* |  |  | 0.999 | 0.976-1.021 | 1.013 | 0.991-1.035 |
| Number of emergency hospitals* |  |  | 1.017 | 0.984-1.051 | 1.010 | 0.978-1.043 |
| Workers in primary industry (%) |  |  |  |  | 1.036 | 1.005-1.067 |
| Workers in secondary industry (%) |  |  |  |  | 1.033 | 1.011-1.056 |
| Bachelor's degree or above (%) |  |  |  |  | 0.921 | 0.900-0.944 |
| WAIC（chain 1） | 9004.5 | | 9008.0 | | 8956.4 | |
| Moran’s I (chain 1) | -0.016 | | -0.015 | | -0.008 | |

PWSAI; population-weighted spatial accessibility index, PWTT, population-weighted travel time to the nearest MT capable hospital, WAIC; Watanabe–Akaike information criterion, Cr; credible interval, RR; relative risk, *per 10000 population.

Model 1; included spatial accessibility factors, Model 2; Model 1 + medical resource factors, Model 3; Model 2 + socioeconomic factors.

Table S3 Sensitivity analysis in main analysis (Model 3) using two distinct priors.

|  | Priors (1, 0.1) | | | Priors (0.5, 0.005) | | |
| --- | --- | --- | --- | --- | --- | --- |
|  | RR | 2.5%Cr | 97.5%Cr | RR | 2.5%Cr | 97.5%Cr |
| PWSAI | 1.012 | 0.993 | 1.032 | 1.012 | 0.994 | 1.031 |
| PWTT (hour) | 1.013 | 0.989 | 1.037 | 1.013 | 0.988 | 1.036 |
| Number of physicians* | 1.021 | 0.996 | 1.013 | 1.001 | 0.988 | 1.013 |
| Number of hospitals* | 1.015 | 0.998 | 1.046 | 1.022 | 0.998 | 1.047 |
| Number of clinics* | 1.015 | 0.998 | 1.032 | 1.015 | 0.998 | 1.032 |
| Number of emergency hospitals* | 1.003 | 0.978 | 1.029 | 1.003 | 0.978 | 1.028 |
| Workers in primary industry (%) | 1.011 | 0.986 | 1.035 | 1.010 | 0.986 | 1.034 |
| Workers in secondary industry (%) | 1.027 | 1.009 | 1.046 | 1.028 | 1.009 | 1.047 |
| Bachelor's degree or above (%) | 0.917 | 0.899 | 0.936 | 0.916 | 0.898 | 0.934 |

PWSAI; population-weighted spatial accessibility index, PWTT, population-weighted travel time to the nearest MT capable hospital, RR; relative risk, Cr; credible interval. *Per 10000 population
